# Assessing cost-effectiveness in oncology treatment sequences: a review of pathway modeling methods for health technology assessment

**DOI:** 10.1017/S0266462326103869

**Published:** 2026-06-24

**Authors:** Lucy Beggs, Kusal Lokuge, Ayman Sadek, Nicky J. Welton, Amy Finnegan, David Nicholls, Lindsay Claxton

**Affiliations:** 1 National Institute for Health and Care Excellence, UK; 2 University of Bristol, UK

**Keywords:** models, economic, evidence synthesis, medical oncology, treatment sequences, methodology

## Abstract

**Objectives:**

Pathway models incorporate multiple decision nodes to assess the most cost-effective sequence or the optimal point of introduction of a new technology within a treatment pathway. Pathway models are particularly useful in disease areas such as oncology, where patients may have several lines of therapy. We aimed to review methodologies for modeling and evidence synthesis in pathway models that evaluate the cost-effectiveness of treatment strategies within oncology.

**Methods:**

We designed a search to identify relevant methodological papers and systematic reviews of oncology pathway models that critique their methodological approaches. We also updated a previous review of studies on methods for evidence synthesis to inform pathway models. Best practice recommendations were extracted and summarized.

**Results:**

Nine and five studies were included on methods for model structures and evidence synthesis, respectively. Key themes related to data requirements, including a preference for patient-level model structures and data from multi-line sources. There was limited guidance on alternative model structures in the absence of patient-level data. Multi-state network meta-analysis with flexible survival models was the most appropriate method identified for evidence synthesis; however, due to data limitations, it may be necessary to conduct separate syntheses at each line of therapy.

**Conclusions:**

Data limitations may reduce the potential benefits of pathway models. Further method development is needed for pathway models in decision spaces where individual patient data and sources that cover multiple lines of treatment are not available.

## Highlights

This systematic literature review summarizes existing methods and frameworks for evidence synthesis and modeling of treatment sequences in oncology. Patient-level model structures using multi-line sources of data are recommended, but our review reveals that there is a lack of guidance on appropriate methods if patient-level data is unavailable. Our summary of existing best practice recommendations and identification of research gaps supports economists developing, and innovating methods for, pathway models.

## Introduction

Health Technology Assessment (HTA) bodies, such as the National Institute for Health and Care Excellence (NICE) in England, produce guidance on the use of interventions based on clinical and cost-effectiveness. Although NICE technology appraisals (TA) may assess single or multiple interventions (1), they typically focus on comparisons of interventions at a single decision point, aligned to the technology’s marketing authorization.

This can lead to development of multiple separate models for the same disease area (2). Each model needs to be scrutinized for decision making due to differences in inputs and assumptions, risking inconsistency (3;4) and potential resource inefficiencies.

One way to address these challenges is to use pathway models (sometimes termed treatment sequence models). For the purposes of this paper, a pathway model is defined as an economic model that incorporates multiple decision points in a treatment pathway, where each decision point is modeled in enough detail to be suitable for decision making in a health technology assessment. Pathway models can therefore be multi-use, with the same model informing different evaluations at individual decision points in a pathway. Pathway models can also be used to assess the optimal treatment sequence or positioning of a new technology within a pathway to help decision makers optimize patient care and resource allocation; this may have particular value for disease areas with multiple lines of treatment such as oncology (5). There has been limited use of pathway models in NICE to date; a single pathway model was recently piloted by NICE for renal cell carcinoma (5).

To support the development of a pathway model for TAs in non-small cell lung cancer (NSCLC), we aimed to review methodologies for pathway models. The review focuses on oncology, which routinely involves multiple lines of treatment and thus lends itself to sequential modeling; however, results may be relevant to other disease areas with multiple lines of therapy.

## Methods

We designed a search to identify (1) papers evaluating approaches for pathway modeling in oncology, and (2) systematic reviews of oncology pathway models that critique their methodological approaches. The search did not aim to identify every individual oncology pathway model, as the focus was on identifying best practice methodology.

The search strategy was designed using four test papers (6–9). Lord et al. (8) had conducted previous work on the use of pathway models, so a 2012 date limit was applied. The search was limited to English-language papers and was run through MEDLINE, Embase, EconLit, and INAHTA in July 2023. Citationchaser was used to retrieve papers citing the included studies.

To identify methodologies for evidence synthesis of relative treatment effects to inform pathway models, an additional search updated the Lewis et al. (9) review of quantitative evidence synthesis methods to assess the effectiveness of sequential treatments. Full details of search strategies are available in the appendix.

The modeling search was updated in November 2023 and both searches were re-run in November 2023 and June 2025.

Results for the methodology search were screened by LB, KL and double-screened by NJW and AS, and results for the evidence synthesis search were screened by AS and NJW, with concordance established via discussions.

Details of included studies were extracted and summarized. This included model structure, best practice recommendations for developing pathway models and choices made in software, and terminology used. Key themes were identified across studies and recommendations were grouped according to theme.

Search strategies and inclusion criteria for each review are included in Supplementary Tables S1–S4.

## Results

For the review of modeling methodology, 358 unique studies were identified. 317 studies were excluded after title and abstract screening, with most being either non-pathway or non-oncology literature. Following the full-text sift, 7 studies (6–8, 10–13) relating to methodological approaches in oncology pathway modeling were included. The search was re-run in June 2025 and a further 468 new studies were identified. A total of 455 were excluded after screening on title and abstract, and 11 were excluded in the full-text sift, leaving 2 additional includes (14;15).

For the review of evidence synthesis methods, of the five papers identified in the Lewis et al review (9) that were on oncology, only one paper (16) met the inclusion criteria. In the initial update of the Lewis et al (9) review, 735 unique studies were identified, of which 3 studies (11;17;18) were included. The search was re-run in June 2025 and a further 169 studies were identified, of which 1 additional study (19) was included, resulting in five included studies (11;16–19).

**Figure 1.**
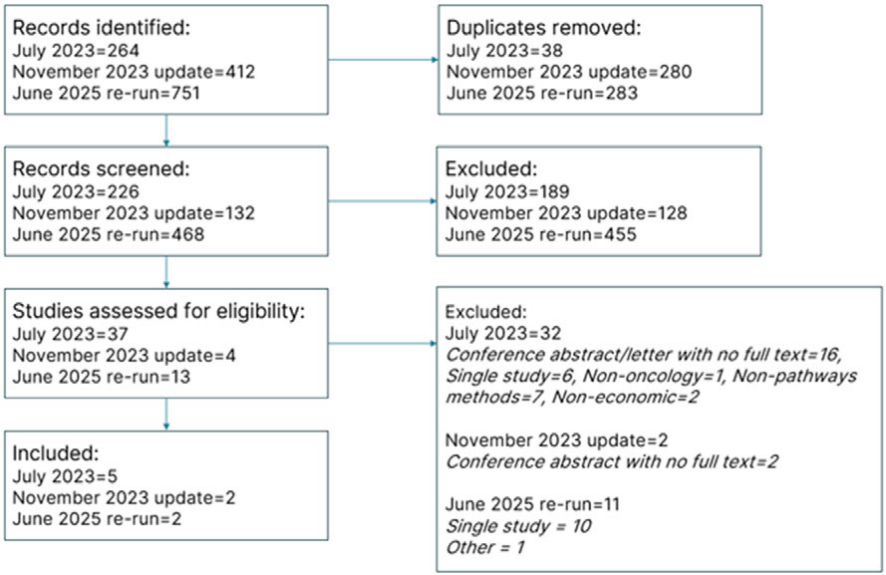
PRISMA diagram for modeling review. Figure 1. long description.

**Figure 2.**
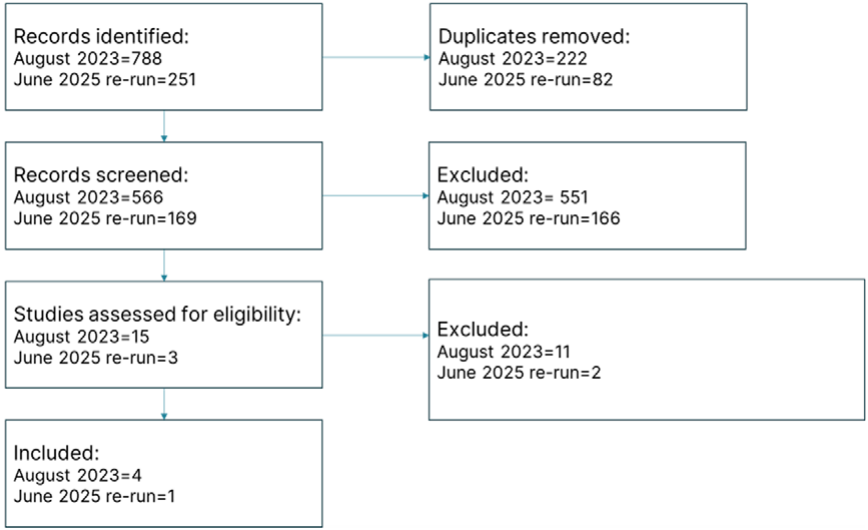
PRISMA diagram for evidence synthesis review. Figure 2. long description.

PRISMA diagrams are shown in Figures 1 and 2.

### Summary of included studies on methodology for pathway models

Huang et al. (6): This study is a systematic review exploring methods for modeling efficacy outcomes in oncology sequence models. It makes the following recommendations: (1) Where possible, models should include progression, discontinuation and death as outcomes; (2) Use of long-term trials with sufficient follow-up to estimate survival for treatment sequences, or in the absence of this, use of individual patient data (IPD) to adjust for differing patient characteristics and outcomes based on position of treatment in sequence; (3) Incorporation of treatment-free intervals in a model framework, ideally informed by IPD or by literature and expert opinion if not available; (4) Use of indirect treatment comparison (ITC) data for each line of treatment in the absence of direct comparative trial data.

Tappenden et al. (7): This study reports a methodological framework for whole-disease modeling, primarily developed for cancer but generalizable to other diseases. The paper outlines a five-stage process for developing and using whole-disease models. Stage one involves understanding the decision problem. Stage two involves model conceptualization via modeling underlying disease and service pathways. Stage three recommends model implementation using patient-level simulation. It also suggests continuous discounting for QALYs and standard discounting for costs, and the use of probabilistic methods for exploring variability and parameter uncertainty. Stages four and five recommend model checking based on Chilcott et al. (20), and consideration of multiple frameworks for decision making.

The framework outlined was later implemented to develop an evaluation for colorectal cancer by Tappenden et al. (10).

Zheng et al. (13): This study is a review of approaches used to model treatment sequences in health economic models in NICE TAs. The study makes the following recommendations: (1) Sequences and treatment switching should be modeled if the selection, efficacy or costs of treatment are affected by prior treatments, or if the decision problem is about positioning within a pathway; (2) Patient-level modeling approaches are preferred if patient characteristics or treatment history affects subsequent treatment, event risks change with time, or there are competing risks; (3) Model validation using clinical expertise and real-world evidence (RWE), and exploration of uncertainty through sensitivity/scenario analyses.

Lord et al. (8): The study describes two models for NICE guidelines on prostate cancer and atrial fibrillation, of which the prostate cancer model meets the inclusion criteria. In its discussion, the paper concludes that modeling complex pathways using discrete event simulation (DES) is less cumbersome and easier to understand than Markov or decision tree models, but recognizes a potential need for upskilling modelers in the skills required for DES. The study highlights that access to patient-level data on characteristics is essential for DES, and suggests the use of audit or registry data.

The authors note the pathway model generated more evidence for decision makers than a single model, but it was unclear whether development of the whole-disease model used fewer resources than multiple single models covering the same pathway, and whether such complex evidence could be usefully interpreted by decision makers. The authors also highlight the risks that errors could be propagated throughout the evaluation of multiple different decisions, and that re-running analyses holds an increased computational burden.

The study makes good practice recommendations around: (1) Clarity about the boundaries of the model; (2) Establishing whether the model pathway reflects recommended or current practice; (3) Capturing the interaction between disease progression over time with the service pathway; (4) The importance of visual and textual representation of the pathway for model users, developers and decision makers; (5) The need for justifications of simplifying assumptions; (6) Recognition of potential inconsistencies between bodies of evidence that inform different sections of the model.

The paper suggests further research may be needed on calibration methods to infer missing or unobservable parameters, methods for testing the internal and external validity of guidelines based on pathway models, and standard templates for presenting models.

Jansen et al. (11): This paper describes an open-source patient-level model comparing the cost-effectiveness of multiple treatment sequences for NSCLC.

Methodological discussion in the paper concentrates on the value and feasibility of developing an open-source model, rather than pathway modeling methods. Additional hand-searching found model documentation (21) outlining the model structure on GitHub.

The model was developed in R, with authors noting that the development of complex models may not be feasible in Microsoft Excel. Other cited advantages of R include the ability to contain parameter estimation and simulation in the same environment, ease of running probabilistic sensitivity analysis, version control, reproducibility of scripts, and transparency.

Jin et al. (12): This study is a systematic review and critical appraisal of whole-disease models. It argues that patient-level models are more appropriate than whole-disease models as they allow for the risk of events to change over time depending on patient characteristics and previous events. However, patient-level models require more computational time.

The study makes four recommendations: (1) Reporting of whole-disease models should follow the CHEERS checklist (22); (2) The appropriateness of alternative modeling methods should be assessed and the chosen method justified; (3) Costs and outcomes of important adverse events should be incorporated; (4) Uncertainty should be explored with extensive deterministic and probabilistic sensitivity analyses.

Wang et al. (14): This study presents results from a modified Delphi panel about multi-use disease models (MUDMs), using a definition that covers pathway models intended for use in multiple HTAs. Participants considered the development, validation, and maintenance of models. Issues ranked as most important were model transparency, transferability, complexity, and the role of stakeholders. The article highlights that model scopes should balance feasibility with applicability, and functions enabling model maintenance should be incorporated from the start of model development. It also discusses the overlap and differences between MUDMs, whole-disease models, and reference models.

Viola et al. (15): This paper updates the review by Zheng et al. (13) and argues the importance of using optimally cost-effective treatment sequences as comparators when evaluating new treatment sequences. A simplified model demonstrates that including a non-cost-effective treatment in a comparator sequence changes the cost-effectiveness of the sequence being evaluated.

### Summary of included studies on methodology for evidence synthesis of relative effects to inform pathway models

Stenner et al. (16): This study combines observational data comparing two different sequences (sorafenib followed by sunitinib or vice versa) for metastatic colorectal cancer from five Swiss centers. However, the authors provided few details on the methods of synthesis.

Diaby et al. (17) and Handorf et al. (19) incorporated evidence from multiple treatments, but undertook separate analyses for each treatment and line of therapy, rather than using evidence synthesis methods such as network meta-analysis (NMA). Diaby et al. (17) fitted a range of parametric models for progression-free survival (PFS) and overall survival (OS), including proportional hazards and accelerated failure time models, to parameterize four treatment sequences over three lines of therapy for HER2+ metastatic breast cancer. Handorf et al. (19) populated a micro-simulation model comparing two treatment sequences over two lines of therapy for metastatic castration-sensitive prostate cancer patients. They developed a novel adaptation of the Guyot et al. algorithm (23) to obtain digitized OS, PFS, and time-to-progression (TTP) curves from trials, allowing transition probabilities to be estimated accounting for competing risks. Trials were mostly chemotherapy-naïve patients treated at first line (24;25), so Handorf et al. (19) proposed a calibration approach to adjust curves to reflect a second-line population.

Jansen et al. (11): This study estimated multi-state NMA models at each line of therapy using a mixture of Weibull models and flexible fractional polynomial models to allow for time-varying hazard ratios. A multinomial likelihood is given for the number of patients progression-free, progressed, and dead at each non-overlapping interval extracted from Kaplan–Meier curves. The approach is novel and relevant for pathway models, although data availability is an issue. Incerti and Jansen (21) only fitted the multi-state NMA models for first-line treatments, where they needed to adopt some strong assumptions: no treatment effect on the second shape parameter for the fractional polynomial for TTP; pre-progression mortality was assumed independent of treatment; and the shape parameter for post-progression mortality does not depend on treatment. At second line, only absolute effects were estimated in a meta-analysis for each treatment independently due to a lack of data.

Schettini et al. (18) undertook Bayesian NMAs assuming proportional hazards for PFS, overall response rate (ORR), and OS, at first line and later lines along the treatment pathway for metastatic triple-negative breast cancer. The authors considered each line separately and did not compare sequences or estimate a pathway model using the results. They considered patients with different genetic mutations as separate subgroups where possible, although some studies reported aggregated results only, and some studies reported subgroup results aggregated over the treatment line.

## Discussion

One of the strongest themes that emerged from the review was that data limitations may present a barrier to the full realization of the benefits of pathway models. To characterize effects of prior treatment requires access to IPD or subgroup data based on treatment history, and the absence of this data may restrict evidence synthesis to individual decision nodes. Similarly, preferred model structures rely on access to IPD. Ideally, data would come from sources that cover multiple lines of treatment, as combining effectiveness estimates from multiple trials in a pathway model may lead to bias. Furthermore, OS trial data may only be suitable for modeling the last line of treatment due to confounding.

Further themes relate to software and nomenclature.

### Requirement of IPD for evidence synthesis and patient-level model structures

One benefit of pathway models over single decision point models is the potential to capture how prior treatment and patient characteristics influence subsequent treatment effects. However, this typically requires IPD or subgroup data based on treatment history. Although manufacturers may have treatment history information from their own trials, statistical power to detect effects is likely limited, and they are unlikely to have access to such information from other trials in the pathway. Availability of IPD is likely to depend on data-sharing incentives and should be considered when conceptualizing models. IPD can be constructed with sufficient data about patient characteristics to allow simulation of a data set, but this level of detail is rarely reported and only likely to be acquired through primary analysis of a real-world data set which may be too time-consuming for live HTAs.

Of the evidence synthesis methodologies identified, the most appropriate is the multi-state NMA approach proposed by Incerti and Jansen (21), because it can capture treatment effects on progression and survival at each line of therapy. However, in practice, the lack of IPD limits the flexibility of the fractional polynomial models that can be fitted, and as a result it may be necessary to model each decision-node separately.

Several studies (7;8;10;11;13) used or recommended patient-level model structures when developing pathway models, arguing that they are more flexible than cohort models and better suited to characterizing complex pathways. However, as with evidence synthesis, the availability of IPD can limit the feasibility (8). Patient-level simulations can also increase computational time, especially when doing probabilistic sensitivity analyses (12). No studies gave recommendations for the appropriate model structure if IPD is unavailable, reflecting a research gap.

### Preference for data from sources that cover multiple lines of treatment

Ideally, pathway models would be informed by trials that cover multiple lines of treatment (6;13). Common trial inclusion criteria mean later-line treatment trials often enroll healthier patients who have better performance than patients who receive them as subsequent treatments in trials of earlier lines. Failure to adjust for this when combining estimates from multiple trials may overestimate later-line treatment effects and bias cost-effectiveness results. Furthermore, earlier trials with fewer treatments available are unlikely to capture contemporary estimates of effectiveness. Zheng (13) highlights a comparison of two scenarios in a NICE appraisal (26), one which took the third-line treatment efficacy estimates from a third-line trial, and one which took the third-line estimates from follow-up from a trial of an earlier line of treatment. The former scenario had more favorable survival estimates.

However, studies (6;11;13) recognize that single-trial efficacy estimates for multiple lines of treatments are uncommon. Only 3 of 46 economic models identified by Huang et al. (6) used treatment sequence estimates from a single multi-line trial; most estimated efficacy from multiple separate sources. Even when subsequent treatment data are collected, short trial durations limit the amount and quality of this evidence (13).

Huang et al. (6) suggest that if IPD were available for multiple treatment lines, evidence from earlier lines of treatment could be used to develop a prognostic model to adjust the population characteristics of patients receiving later-line treatments. However, their review did not identify any models that did this.

Real-world data, such as retrospective cohort data or registry data, could be explored as alternative sources of clinical data (6;13). However, caution would have to be exercised to overcome other forms of bias associated with observational evidence (27). Further guidance is needed on the optimal way to adjust for selection bias when pathway models are informed by multiple trials due to the absence of multi-line sources of evidence or IPD.

### Suitability of OS trial data

OS data from trials will include patients who had a range of post-progression, and in multi-center international trials, these could vary substantially. This raises potential confounding issues, as OS data captures pre- and post-progression mortality. As a result, OS trial data may only be informative for the last line of therapy, and other sources, such as cancer registries, may provide more relevant information on pre-progression mortality and mortality for patients who do not proceed to have all lines of therapy.

### Software

Models identified in this review were built using several different software packages. Zheng et al. (13) include models built in Microsoft Excel, TreeAge, R, and Arena. Models developed by Tappenden et al. (10) and Lord et al. (8) used SIMUL8, while the IVI-NSCLC model (11) was developed in R. Jansen et al. (11) comments extensively on the merits of script-based languages over spreadsheet software for simulation modeling, arguing that the latter would not be able to implement semi-Markov patient-level simulations efficiently. Both Jansen et al (11) and Zheng et al (13) comment on the potential to use script-based languages to increase efficiencies and reduce computational burden in complex modeling. Cited advantages of R include the ability to contain parameter estimation and simulation in the same environment, ease of running probabilistic sensitivity analysis, reproducibility of scripts, and increased transparency (11;19).

However, there may be operational barriers to using script-based or specialist simulation software for pathway models. In general, modelers should be mindful of users when choosing software to ensure it does not pose a barrier to usefulness, interpretation by decision makers, and critique from stakeholders. Specialist software, such as SIMUL8 and other DES packages, often includes license fees and is not used by all model developers; using this software may require financial investment and upskilling of modelers.

R is frequently used in statistics and data analytics, but has not historically been widely adopted by health economists (11). The advantages of R are becoming more established in the health economics community, but the current lack of familiarity amongst developers and quality assurers means there will likely be a lag to more widespread implementation in HTA agencies.

There is, therefore, still a place for method development in pathway modeling that can be implemented in conventional spreadsheet software.

### Lack of consistency in nomenclature for model structures

As part of the scoping process for the development of an NSCLC pathway model, academic and industry stakeholders identified terms to describe pathway models: “pathway models,” “whole-disease models,” “core/core-disease models,” and “treatment sequence models.”

Our search revealed inconsistent terminology for economic pathway model structures. Several papers used “pathway” and “treatment sequence” for non-economic models of disease pathways, clinical care pathways, or in-depth costings analyses. Some economic models labelled as pathway models were actually conventional three-state oncology models, where subsequent treatments were not modeled in detail (6).

Although IVI-NSCLC (11) is a pathway model, its abstract does not include predefined model structure terms and was only identified by the phrase “model sequential treatment.” The most detailed documentation is a non-peer-reviewed GitHub file (21) identified by hand-searching (although challenges of identifying this document relate primarily to its open-source nature and are independent of the model structure).

Terminology is discussed by Wang et al (14), with the Delphi panel preferring “multi-use disease model.” Due to their comprehensive scope, pathway models may be useful multi-use reference models for disease areas; they could provide a standardized framework through which new and existing interventions can be evaluated, allow unbiased comparative analysis between interventions, and reduce model development times (28). Although there is overlap, being a multi-use or reference model is neither a necessary or sufficient condition for treatment-sequencing pathway models; thus, a distinct descriptor remains needed.

Varied terminology means searches for methodological developments risk retrieving an unmanageable number of records or excluding relevant papers not indexed with common terms.

### Strengths

This review enhances understanding of pathway modeling and evidence synthesis methodologies, and highlights a priority area for future methodological development: guidance on best practice for pathway modeling in the absence of IPD and multi-line data.

Medicine innovations mean there are often an increasing number of treatment options at single decision points, so decision makers may want to explore sequencing treatments as a way of balancing access to treatments with value for money. As these multi-comparator decision spaces become more common, the need for a flexible range of modeling approaches for treatment sequences becomes increasingly important. In this context, a key advantage of this review is our consideration of operational feasibility for real-world HTA settings. Key implications for HTA agencies are that pathway models present an opportunity for multi-use modeling, which may make efficient use of HTA bodies’ resources, but their feasibility and value may be diminished without access to IPD, multi-line, and registry data; the former may require consideration of data-sharing incentives. Proceeding without ideal data may be possible, but it limits opportunities for evidence synthesis to characterize conditionalities between decision nodes; further methodological guidance is also needed on appropriate model structures. Decision makers should be aware that these data limitations increase the risk of bias and decision uncertainty. From an operational perspective, script-based or specialist software may provide better flexibility for pathway modeling, but consideration should be given to the familiarity of developers and users

### Limitations

The review was designed to support the development of a pathway model for advanced NSCLC and focused on oncology. However, there may be methods developed in a non-oncology setting that could have applicability to oncology modeling that would not be captured by the search.

The variation in terms used to describe pathway models meant relevant methods papers may not have been identified in this review due to using nomenclature unfamiliar to the authors. The risks of this have been mitigated through discussion of terms used to describe pathway models with stakeholders, but there is still potential for papers using different terms to have been missed.

Our study focuses on how to best implement pathway models, but as model development time and resources are finite, exploration is needed to characterize when and why a pathway approach should be taken over simpler approaches. Pathway models’ potential use as reference models is another area that could be explored further.

## Conclusions

Our review highlights the data requirements for pathway models, which limit their applicability in practice. Further guidance is required on: (1) methods for evidence synthesis and model structure in the absence of IPD, (2) optimal adjustment of data in the absence of data sources that cover multiple lines of treatment.

## Supporting information

10.1017/S0266462326103869.sm001 Beggs et al. supplementary material Beggs et al. supplementary material

## Supplementary material

The supplementary material for this article can be found at http://doi.org/10.1017/S0266462326103869.

## Long descriptions

### Figure 1. Long description

The flowchart consists of four main vertical stages on the left and three exclusion boxes on the right.

1. Records identified: July 2023 equals 264, November 2023 update equals 412, June 2025 re-run equals 751. An arrow points right to Duplicates removed: July 2023 equals 38, November 2023 update equals 280, June 2025 re-run equals 283.

2. Records screened: July 2023 equals 226, November 2023 update equals 132, June 2025 re-run equals 468. An arrow points right to Excluded: July 2023 equals 189, November 2023 update equals 128, June 2025 re-run equals 455.

3. Studies assessed for eligibility: July 2023 equals 37, November 2023 update equals 4, June 2025 re-run equals 13. An arrow points right to a detailed Excluded box:

- July 2023 equals 32 (Conference abstract/letter with no full text equals 16, Single study equals 6, Non-oncology equals 1, Non-pathways methods equals 7, Non-economic equals 2).

- November 2023 update equals 2 (Conference abstract with no full text equals 2).

- June 2025 re-run equals 11 (Single study equals 10, Other equals 1).

4. Included: July 2023 equals 5, November 2023 update equals 2, June 2025 re-run equals 2.

Navigate back to Figure 1..

### Figure 2. Long description

The flowchart consists of four primary vertical stages with three horizontal exclusion branches.

1. Identification stage at the top left. Records identified: August 2023 = 788, June 2025 re-run = 251. An arrow points right to Duplicates removed: August 2023 = 222, June 2025 re-run = 82.

2. Screening stage. Records screened: August 2023 = 566, June 2025 re-run = 169. An arrow points right to Excluded: August 2023 = 551, June 2025 re-run = 166.

3. Eligibility stage. Studies assessed for eligibility: August 2023 = 15, June 2025 re-run = 3. An arrow points right to Excluded: August 2023 = 11, June 2025 re-run = 2.

4. Inclusion stage at the bottom. Included: August 2023 = 4, June 2025 re-run = 1.

Navigate back to Figure 2..
