# Supplementary material for: Assessing cost-effectiveness in oncology treatment sequences: a review of pathway modeling methods for health technology assessment
Source: Int J Technol Assess Health Care. 2026 Jun 24;42(1):e60. doi: 10.1017/S0266462326103869 (PMC13440441; doi:10.1017/S0266462326103869)
Supplement: Supplementary file 1 [file S0266462326103869sup001.docx]

# Supplementary files

## *Search strategies*

Table 1: Strategy for modelling search

**Database: MEDLINE ALL**

Strategy used:

1	exp Medical Oncology/ or exp Integrative Oncology/ or exp Lung Neoplasms/ or exp Neoplasms/ or Oncology Treatment Sequence Model/ (3862765)

2	((lung* or pulmonar* or respirat*) adj2 (cancer* or carcino* or tumo?r* or neoplasm* or adenocarcino* or mass* or malignan*)).ti,ab. (265711)

3	oncolog*.ti,ab. (194599)

4	or/1-3 (3982026)

5	((pathway* or whole* or core* or system or systems or sequenc*) adj1 (disease* or treatment* or algorithm* or decision*) adj1 (model* or method* or framework*)).ti,ab,kw,kf. (167)

6	MAPguide*.ti,ab,kf,kw. (2)

7	or/5-6 (168)

8	4 and 7 (36)

9	8 and (2012* or 2013* or 2014* or 2015* or 2016* or 2017* or 2018* or 2019* or 202*).dt,ed. (25)

10	limit 9 to english (25)

11	10 not (animals/ not humans/) (24)

12	limit 11 to (letter or historical article or comment or editorial or news or case reports) (1)

13	11 not 12 (23)

**Notes:**

Oncology Treatment Sequence Model/ Is not a MeSH term, however it does pick up a test paper that is still indexed as pubmed-not medline.

**Database: Embase**

Strategy used:

1	oncology/ or integrative oncology/ or exp lung tumor/ or exp neoplasm/ or Oncology Treatment Sequence Model/ (5595581)

2	((lung* or pulmonar* or respirat*) adj2 (cancer* or carcino* or tumo?r* or neoplasm* or adenocarcino* or mass* or malignan*)).ti,ab. (400320)

3	oncolog*.ti,ab. (370998)

4	or/1-3 (5721635)

5	((pathway* or whole* or core* or system or systems or sequenc*) adj1 (disease* or treatment* or algorithm* or decision*) adj1 (model* or method* or framework*)).ti,ab,kw,kf. (528)

6	MAPguide*.ti,ab,kf,kw. (5)

7	or/5-6 (532)

8	4 and 7 (145)

9	8 and (2012* or 2013* or 2014* or 2015* or 2016* or 2017* or 2018* or 2019* or 202*).dc. (120)

10	limit 9 to english language (118)

11	nonhuman/ not (human/ and nonhuman/) (5338883)

12	10 not 11 (116)

**Database: Econlit**

Strategy used:

1	((lung* or pulmonar* or respirat*) adj2 (cancer* or carcino* or tumo?r* or neoplasm* or adenocarcino* or mass* or malignan*)).ti,ab. (193)

2	oncolog*.ti,ab. (116)

3	or/1-2 (303)

4	((pathway* or whole* or core* or system or systems or sequenc*) adj1 (disease* or treatment* or algorithm* or decision*) adj1 (model* or method* or framework*)).ti,ab. (3)

5	MAPguide*.ti,ab. (0)

6	or/4-5 (3)

7	3 and 6 (0)

8	limit 7 to english (0)

**Database: INAHTA**

Strategy used:

Line	Search query	Hits

#1	"Medical Oncology"[mhe]	25

#2	"Integrative Oncology"[mhe]	0

#3	"Lung Neoplasms"[mhe]	402

#4	"Neoplasms"[mhe]	3917

#5	oncolog*	237

#6	((lung* or pulmonar* or respirat*) AND (cancer* or carcino* or tumor* or tumour* or neoplasm* or adenocarcino* or mass* or malignan*))	496

#7	#6 OR #5 OR #4 OR #3 OR #2 OR #1	4072

#8	((pathway* or whole* or core* or system or systems or sequenc*) AND (disease* or treatment* or algorithm*) AND (model* or method* or framework*))	494

#9	MAPguide*	1

#10	#8 OR #9	494

#11	#7 AND #10	99

#12	Limit to English language	56

#13	Limit to 2012-2023	31

**Database: Citation chaser**

Strategy used:

The four DOIs from the test papers provided by the request form were searched:

doi.org/10.1016/j.jval.2012.07.008

doi.org/10.3310/hta17580

doi.org/10.1007/s40273-020-00980-w

doi.org/10.1007/s40273-021-01113-7

Notes:

Forward citation searches were performed using [Citation chaser](https://estech.shinyapps.io/citationchaser/)

Table 2: Strategy for evidence synthesis search

**Database: MEDLINE**

Database: Ovid MEDLINE(R) ALL <1946 to August 16, 2023>

Search Strategy:

--------------------------------------------------------------------------------

1	Meta-analysis as topic/

2	Meta-analysis/

3	meta-analysis.pt.

4	(meta-analys* or metaanalys*).sh.

5	(meta-analys* or metaanalys*).ti,ab.

6	meta-regression.ti,ab.

7	systematic review.ti,ab.

8	Cochrane.ti,ab.

9	health care costs/

10	cost-benefit analysis/

11	Cost benefit analys*.ti,ab.

12	((cost effectiveness or cost utility or cost consequence*) adj2 analys*).ti,ab.

13	Economic evaluation.ti,ab.

14	sensitivity analys*.ti,ab.

15	Health technology assessment.ti,ab.

16	Guideline.pt.

17	Practice guideline.pt.

18	Clinical guideline*.ti,ab.

19	NICE guid*.ti,ab.

20	Comparative effectiveness research.ti,ab.

21	or/1-20

22	(Sequen* adj2 (treatment* or therap* or drug* or medication* or regimen* or intervention* or biologic*)).ti,ab.

23	21 and 22

24	exp Medical Oncology/

25	exp Neoplasms/

26	(oncolog* or cancer* or carcinoma* or malignan* or neoplas* or tumour* or tumor* or adenocarcin* or adeno-carcin* or sarcoma* or choriocarcinoma* or chorioncarcinoma* or dysgerminoma* or seminoma* or teratoma* or teratocarcinoma* or terato-carcinoma* or cystadenocarcin* or fibrosarcoma* or fibrosarcoma* or rhabdomyosarcoma* or rhabdo-myosarcoma* or rhabdosarcoma* or rhabdo-sarcoma* or leiomyosarcoma* or leio-myosarcoma* or carcinosarcoma* or carcino-sarcoma* or granulosa*).ti,ab.

27	or/24-26

28	23 and 27

29	limit 28 to english language

30	limit 29 to ed=20130801-20230817

31	limit 29 to dt=20130801-20230817

32	30 or 31

**Database: EMBASE**

1	"meta analysis (topic)"/

2	meta analysis/

3	meta-analysis.pt.

4	(meta-analys* or metaanalys*).sh.

5	(meta-analys* or metaanalys*).ti,ab.

6	meta-regression.ti,ab.

7	systematic review.ti,ab.

8	Cochrane.ti,ab.

9	"health care cost"/

10	"cost benefit analysis"/

11	Cost benefit analys*.ti,ab.

12	((cost effectiveness or cost utility or cost consequence*) adj2 analys*).ti,ab.

13	Economic evaluation.ti,ab.

14	sensitivity analys*.ti,ab.

15	Health technology assessment.ti,ab.

16	Guideline.pt.

17	Practice guideline.pt.

18	Clinical guideline*.ti,ab.

19	NICE guid*.ti,ab.

20	Comparative effectiveness research.ti,ab.

21	or/1-20

22	(Sequen* adj2 (treatment* or therap* or drug* or medication* or regimen* or intervention* or biologic*)).ti,ab.

23	21 and 22

24	exp oncology/

25	exp neoplasm/

26	(oncolog* or cancer* or carcinoma* or malignan* or neoplas* or tumour* or tumor* or adenocarcin* or adeno-carcin* or sarcoma* or choriocarcinoma* or chorioncarcinoma* or dysgerminoma* or seminoma* or teratoma* or teratocarcinoma* or terato-carcinoma* or cystadenocarcin* or fibrosarcoma* or fibrosarcoma* or rhabdomyosarcoma* or rhabdo-myosarcoma* or rhabdosarcoma* or rhabdo-sarcoma* or leiomyosarcoma* or leio-myosarcoma* or carcinosarcoma* or carcino-sarcoma* or granulosa*).ti,ab.

27	or/24-26

28	23 and 27

29	limit 28 to english language

30	limit 29 to dc=20130801-20230817

**Database: Econlit**

Database: Econlit <1886 to August 03, 2023>

Search Strategy:

--------------------------------------------------------------------------------

1	(meta-analys* or metaanalys*).ti,ab.

2	meta-regression.ti,ab.

3	systematic review.ti,ab.

4	Cochrane.ti,ab.

5	Cost benefit analys*.ti,ab.

6	((cost effectiveness or cost utility or cost consequence*) adj2 analys*).ti,ab.

7	Economic evaluation.ti,ab.

8	sensitivity analys*.ti,ab.

9	Health technology assessment.ti,ab.

10	Clinical guideline*.ti,ab.

11	NICE guid*.ti,ab.

12	Comparative effectiveness research.ti,ab.

13	or/1-12

14	(Sequen* adj2 (treatment* or therap* or drug* or medication* or regimen* or intervention* or biologic*)).ti,ab.

15	13 and 14

16	(oncolog* or cancer* or carcinoma* or malignan* or neoplas* or tumour* or tumor* or adenocarcin* or adeno-carcin* or sarcoma* or choriocarcinoma* or chorioncarcinoma* or dysgerminoma* or seminoma* or teratoma* or teratocarcinoma* or terato-carcinoma* or cystadenocarcin* or fibrosarcoma* or fibrosarcoma* or rhabdomyosarcoma* or rhabdo-myosarcoma* or rhabdosarcoma* or rhabdo-sarcoma* or leiomyosarcoma* or leio-myosarcoma* or carcinosarcoma* or carcino-sarcoma* or granulosa*).ti,ab.

17	15 and 16

18	limit 17 to yr=2013-2023

## *Inclusion criteria for reviews*

Table 3: Inclusion criteria for modelling review

| ***Inclusion criteria*** | ***Exclusion criteria*** |
| --- | --- |
| - Systematic reviews of oncology pathway models - Methodological papers discussing oncology pathway models (involving substantial discussion contributing to methods development/critique rather than solely describing the methods used in the model) - Single oncology pathway models with substantive discussion about methodological approach | - Non-English language - Conference abstract with no published paper - Letters, responses - Non-comparative cost-effectiveness studies - Non-oncology pathway models - Non-pathway models (for examples, typical TA models that don’t model later lines of treatment explicitly) - Single oncology pathway models that do not have a substantive discussion about methodological approach |

Table 4: Inclusion criteria for evidence synthesis review

| ***Inclusion criteria*** | ***Exclusion criteria*** |
| --- | --- |
| - Methodological papers discussing quantitative evidence synthesis of effectiveness of sequential treatments in oncology contributing to methods development - Applications of evidence synthesis in oncology with substantive discussion about methodological approach for the quantitative evidence synthesis of effectiveness outcomes of sequential treatments | - Non-English language - Conference abstract with no published paper - Letters, responses - Papers exploring evidence synthesis methods for non-oncology treatment pathways - Papers exploring evidence synthesis methods at a single point in a treatment pathway |
